# DNA end resection and its role in DNA replication and DSB repair choice in mammalian cells

**DOI:** 10.1038/s12276-020-00519-1

**Published:** 2020-10-30

**Authors:** Fei Zhao, Wootae Kim, Jake A. Kloeber, Zhenkun Lou

**Affiliations:** 1 Department of Oncology, Mayo Clinic, Rochester, MN 55905 USA; 2 Mayo Clinic Medical Scientist Training Program, Mayo Clinic, Rochester, MN 55905 USA; 3 Department of Molecular Pharmacology and Experimental Therapeutics, Mayo Clinic, Rochester, MN 55905 USA

**Keywords:** Double-strand DNA breaks, Cell signalling

## Abstract

DNA end resection has a key role in double-strand break repair and DNA replication. Defective DNA end resection can cause malfunctions in DNA repair and replication, leading to greater genomic instability. DNA end resection is initiated by MRN-CtIP generating short, 3′-single-stranded DNA (ssDNA). This newly generated ssDNA is further elongated by multiple nucleases and DNA helicases, such as EXO1, DNA2, and BLM. Effective DNA end resection is essential for error-free homologous recombination DNA repair, the degradation of incorrectly replicated DNA and double-strand break repair choice. Because of its importance in DNA repair, DNA end resection is strictly regulated. Numerous mechanisms have been reported to regulate the initiation, extension, and termination of DNA end resection. Here, we review the general process of DNA end resection and its role in DNA replication and repair pathway choice.

## DNA repair: tying up loose ends

Carefully regulated enzymatic processing of the ends of DNA strands is essential for efficient replication and damage repair while also minimizing the risk of genomic instability. Replication and repair depend on a mechanism known as DNA resection, in which enzymes trim back double-stranded DNA ends to leave single-stranded overhangs. Zhenkun Lou and colleagues at the Mayo Clinic in Rochester, USA, have reviewed the various steps involved in the initiation and control of DNA resection. There are multiple different DNA repair processes, and the manner in which resection occurs can determine which of these processes subsequently takes place. The authors note that cancer cells rely heavily on these repair pathways to survive radiotherapy and chemotherapy, and highlight research opportunities that might reveal therapeutically useful vulnerabilities in the resection mechanism.

## Introduction

DNA double-strand breaks (DSBs) are seriously harmful genomic lesions that threaten genomic stability and cell survival. Defective DSB repair is associated with embryonic death, aging, immunodeficiency, neurological disorders, and cancer^1–3^. In response to DSBs, the kinases ATM, ATR, and DNA-PKcs become activated and phosphorylate multiple substrates to initiate the DNA-damage response (DDR), leading to the recruitment of DDR factors to DNA-damage sites, cell cycle arrest, and activation of DNA repair. The two major DSB repair pathways that have been extensively investigated are homologous recombination (HR) and non-homologous end joining (NHEJ). NHEJ functions throughout interphase. Error-free repair by HR requires a homologous sister chromatid as a recombination template and hence is favored in the S and G2 phases.

DNA end resection plays a key role in error-free repair because of its essential role in HR^4^. DNA end resection initially generates 3′ single-stranded DNA (ssDNA), which provides a platform for recruiting HR repair-related proteins and prevents DNA repair by NHEJ^5^. For the initiation of DNA end resection, the CtBP-interacting protein (CtIP) functions together with the MRE11–RAD50–NBS1 (MRN) complex to generate a short ssDNA at the DSB ends. After ssDNA is generated by the CtIP/MRN complex, downstream nucleases and helicases, such as exonuclease 1 (EXO1) or DNA replication ATP-dependent helicase/nuclease DNA replication helicase/nuclease 2 (DNA2) and Bloom syndrome protein (BLM), are recruited to extend the 3′-ssDNA for HR-mediated repair^6,7^. End resection is essential not only for HR repair but also for mediating accurate DNA replication by degrading faulty replication forks and activating the ATR-CHK1 pathway. Another important function for DNA end resection is the regulation of DSB repair pathway choice^8^. Sufficient end resection is important for RPA complex and RAD51 loading, which are essential for homologous recombination and error-free repair. In fact, blocking DNA end resection leads to NHEJ repair.

In this review, we summarize the general process of DNA end resection and highlight the function of DNA end resection in DNA replication and DSB repair pathway choice.

## Initiation of DNA end resection

In mammalian cells, the MRN complex consists of three subunits, MRE11, RAD50, and Nibrin (NBS1). All of these components are established regulators of DNA damage signal transduction and DNA end resection initiation. Following DNA damage, the MRN complex is recruited to DSB sites and binds DNA through the RAD50 globular ABC ATPase head domain, which is a tetramer formed by the RAD50 walker A/B ATP-binding motif and MRE11. The extended coiled-coil tail of RAD50 is important for tethering the complex^9^. This tethering function of RAD50 allows the MRN complex to form bridges between free DNA ends and is an important step in the regulation of DSB signal transduction. ATM dimers and CtIP are then recruited to the DSB by the C terminus and Forkhead-associated (FHA) domain/BRCA1 C terminus (BRCT) domain of NBS1, respectively^10,11^. Last, MRE11 acts as the catalytic subunit for the initiation of end resection through its nuclease activity^4^.

MRE11 contains four N-terminal phosphodiesterase motifs that make up the catalytic domain and two DNA-binding domains in the C-terminal region^12^. MRE11 exhibits both 3′–5′ exonuclease and endonuclease activity in vitro. Both the endonuclease and exonuclease activity of MRE11 have been shown to be important for DNA end resection. During end resection, endonuclease activity generates a nick in double-stranded DNA (dsDNA). Then, the 3′–5′ exonuclease activity generates a 3′-ssDNA overhang from the nick. In vitro experiments have also revealed that the phosphodiesterase motifs of MRE11 are essential for its nuclease activities^13^.

RAD50 is a structural maintenance of chromosome family member that contains two ATP-binding motifs (Walker A and Walker B) that together exhibit ATPase activity^14^. The ATPase activity of RAD50 is important for the nuclease activity of MRE11^15^. A previous publication suggested that the 3′–5′ exonuclease activity of the MRN complex is very limited because RAD50 strongly inhibits the exonuclease activity of MRE11 in the presence of ATP^16^. ATP binding to RAD50 induces the conformation of RAD50-MRE11 to close, and MRE11 then exhibits mainly endonuclease activity. ATP hydrolysis changes the conformation of the RAD50-MRE11 complex to an open state, allowing MRE11 exonuclease activity^17^. Thus, RAD50 acts as a molecular switch for controlling MRE11 endonuclease/exonuclease activities.

NBS1 is an important regulator of the MRN complex^18^. NBS1 contains two adjacent BRCT domains and an FHA domain, which are established phosphorylation residue-binding domains. Thus, NBS1 is regarded as an important adaptor protein for MRN complex function^19^. NBS1 modulates both the DNA binding and nuclease activity of MRE11^20^. The FHA domain of NBS1 binds phosphorylated CtIP, which is important for CtIP recruitment to DSB sites^21^. In addition, the C-terminal domain is important for ATM recruitment^10,22,23^. These characteristics make NBS1 absolutely indispensable in the mammalian system, which differ from that in yeast where the Xrs2/NBS1 subunit is not required for end resection reaction in vitro^24^.

**Fig. 1:**
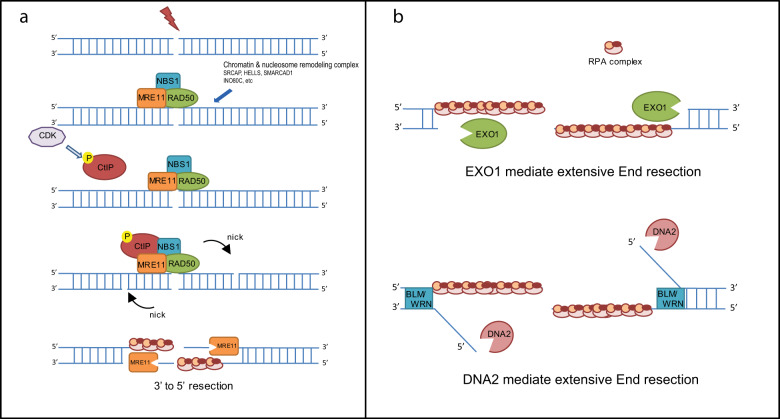
Initiation and extension of DNA end resection. **a** The model of MRN-CtIP and chromatin/nucleosome remodeling proteins in the regulation of DNA end resection initiation. **b** The model of EXO1, DNA2, BLM, WRN and the RPA complex in the regulation of DNA end resection extension.

CtIP, a cofactor for the MRN complex, also has an essential role in DNA end resection initiation. Using an in vitro system containing purified proteins and DNA substrates with blunt ends, in the absence of CtIP, MRN was unable to stimulate DNA end resection^4,25^. The function of CtIP is dependent on CtIP phosphorylation in a CDK target motif at Thr-847, which is important for its association with the MRN complex^26^. Thus, MRE11 endonuclease associated with RAD50, NBS1, and phosphorylated CtIP preferentially generates a short 3′-ssDNA overhang, which is indispensable for extensive DNA end resection and RPA complex loading (Fig. 1a)^18,27^.

## Extension of DNA end resection

The MRN complex and CtIP consistently generate a short (~100 nt) 3′-ssDNA overhang^28^. MRE11 has limited exonuclease activity for producing long 3′-ssDNA overhangs for RPA complex binding. Instead, extensive end resection is performed by EXO1^4^. EXO1 exhibits 5′-to-3′ dsDNA exonuclease and 5′-flap endonuclease activities in vitro^29^. Interestingly, EXO1 prefers dsDNA substrates with a 3′-ssDNA overhang end, which are produced by the MRN complex and CtIP^30,31^. Following MRN-CtIP-mediated end resection initiation, EXO1 is recruited to the DSB site by the MRN complex, and the MRN complex stimulates EXO1 nuclease activity in vitro^24,26^. In addition to EXO1, DNA2 is a key regulator for extensive end resection. DNA2 is a DNA helicase/ssDNA endonuclease that cleaves only free ssDNA ends, with no nuclease effect on dsDNA. It contains a PD-(D/E)XK superfamily nuclease motif and a helicase domain^32^. In vitro DNA2 exhibits 5ʹ- and 3ʹ-endonuclease activities and DNA helicase activity^33,34^. However, extensive end resection is dependent on the nuclease activity of DNA2 combined with the helicase activity of the RecQ helicase BLM^35^. In fact, the DNA helicase BLM is essential for DNA2-mediated extensive end resection. Several studies have shown that DNA2 cleaves ssDNA via BLM-mediated DNA unwinding during end resection^36–38^.

In addition to BLM, Werner syndrome ATP-dependent helicase (WRN), another RecQ family helicase, also functions in DNA end resection in parallel to BLM^39^. Loss-of-function mutations in BLM and WRN cause Bloom and Werner syndromes, respectively^40^. Patients with these syndromes show developmental problems, premature aging, increased genomic instability, and elevated tumorigenesis^41^. However, the distinct roles of BLM and WRN in DNA end resection are still not clearly understood.

Even though EXO1 and the DNA2/BLM/WRN complex show nuclease and DNA helicase activity, they are insufficient to initiate DNA end resection in the absence of the MRN complex^37^. Both WRN-DNA2 and BLM-DNA2 require a short 3′-ssDNA overhang to efficiently extend DNA end resection in vitro, which is in agreement with the current model showing that the initial 5′-end trimming is performed by the MRN complex and CtIP, while EXO1 and DNA2/BLM/WRN are critical for more-extensive resection^25,36^. The RPA complex is also important for promoting the helicase activity of BLM in part by coating unwound ssDNA strands and regulating DNA2 nuclease activity by blocking its 3′–5′ exonuclease activity^34,42^. RPA depletion eliminates long-range extensive DNA end resection and leads to the loss of the 3′-ssDNA overhang ends generated by MRN-CtIP^25^. Thus, EXO1, DNA2, BLM, WRN and the RPA complex constitute the minimal complex that can carry out long-range extensive DNA end resection (Fig. 1b)^36^.

In addition to the DNA end resection machineries (MRN-CtIP, EXO1/DNA2-BLM, and RPA), multiple chromatin remodeling proteins have been reported to regulate the initiation or extension of DNA end resection by relaxing chromatin and thus facilitating access of the core end resection regulators to the broken DNA ends. Dong et al.^43^ reported that the human SNF2-related CBP activator protein (SRCAP) chromatin remodeling complex, which consists of four subunits, SRCAP, ZNHIT1, Arp6, and YL-1, promotes DNA end resection by enhancing CtIP recruitment and chromatin decondensation in an ATPase-dependent manner. Similarly, lymphoid-specific helicase (also known as SMARCA6, LSH, or PASG)^44^, a SNF2-like chromatin remodeling ATPase, was also reported to promote DNA end resection by facilitating the accumulation of CtIP at IR-induced foci. The nucleosome remodeling enzyme, SWI/SNF-related, matrix-associated actin-dependent regulator of chromatin, subfamily A containing DEAD/H box 1 (SMARCAD1, Fun30 in yeast) decreases histone–DNA interactions in nucleosomes flanking DSBs and promotes ssDNA production by the EXO1/DNA2 resection machineries^45,46^. INO80 complex subunit C (INO80C), another nucleosome remodeler involved in the DSB response, shows two distinct functions during HR: promoting DNA end resection and forming the RAD51 presynaptic filament in both yeast and mammalian cells^47,48^.

## Termination of DNA end resection

Proper initiation of end resection is essential for HR-mediated DNA repair. However, uncontrolled end resection also threatens genomic stability, as hyperresection can cause mutational recombination through microhomology-mediated end joining or single-strand annealing, leading to the loss of genetic information^49^. Moreover, unlimited end resection may also reduce RPA recycling efficiency in cells, leading to increased ssDNA exposure, replication fork collapse and genomic instability^50^. Therefore, mechanisms must terminate end resection when the length of ssDNA is sufficient for HR repair, because unlimited extensive end resection is toxic to cells.

**Fig. 2:**
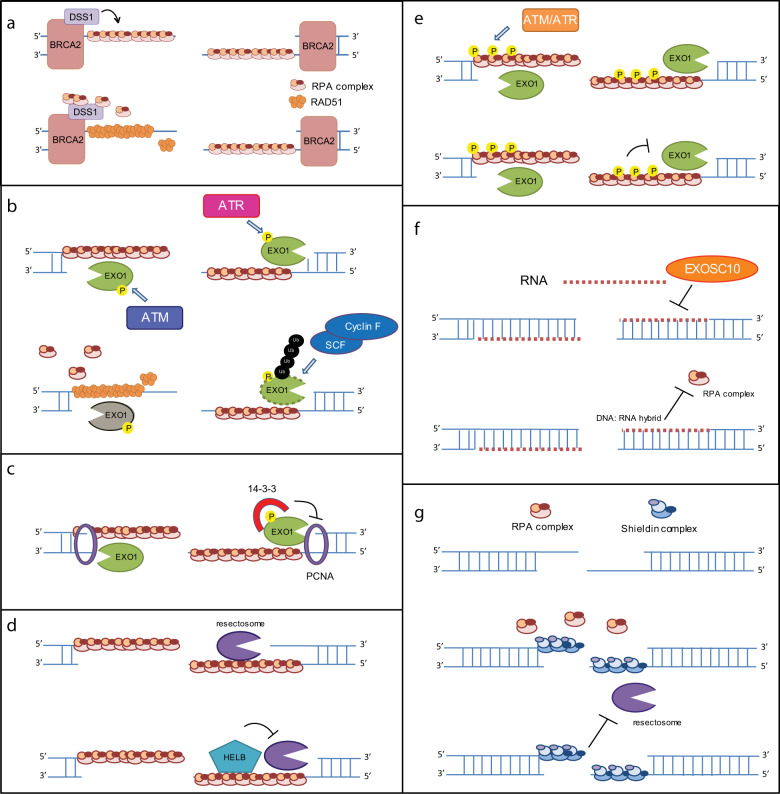
Current models of DNA end resection termination. The regulatory mechanism of DNA end resection termination by BRCA2-DSS1-RAD51 (**a**), EXO1 phosphorylation/ubiquitination (**b**), 14-3-3 (**c**), HELB (**d**), RPA phosphorylation (**e**), EXOSC10 (**f**) and the shieldin complex (**g**).

Although the regulation of DNA end resection termination is still not well studied, several mechanisms have been suggested. Under physiological conditions, end resection is terminated by RAD51-RPA switching. This progress is regulated by BRCA2-DSS1. DSS1 is a small (70 residues) and highly acidic protein that functions by ssDNA mimicry to remove RPA from real ssDNA. Then, RAD51 is recruited by BRCA2, to finish completing the switch (Fig. 2a)^51^.

**Fig. 3:**
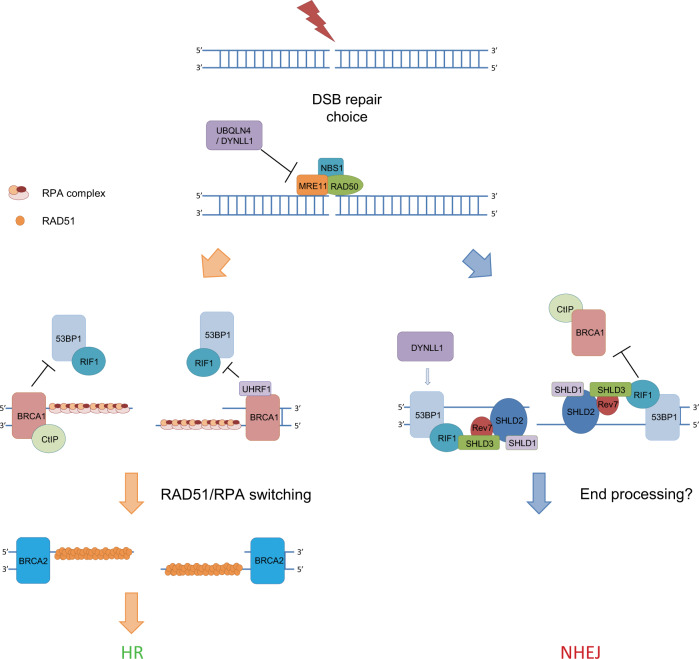
DNA end resection and DSB repair choice. The current view of regulators involved in DNA end resection and DSB repair choice.

Numerous publications show that end resection can be terminated by targeting end resection-regulating proteins. In mammals, EXO1 is rapidly degraded by the Skp1-Cullin1-F-box family of ubiquitin ligases in a proteasome-dependent manner soon after DSB induction^52^. ATR inhibition attenuates EXO1 degradation following DNA damage, suggesting that ATR-mediated EXO1 phosphorylation further promotes EXO1 degradation (Fig. 2b)^52^. Another study shows that ATM-mediated phosphorylation of EXO1 appears to regulate the activity of EXO1 following end resection, promoting RPA disassociation and the completion of HR repair (Fig. 2b)^53,54^. Together, these observations suggest that DNA damage-induced phosphorylation of EXO1 attenuates its function to terminate resection^55^. Several publications also report that 14-3-3 disrupts the EXO1–PCNA interaction following DNA damage, thus attenuating EXO1 exonuclease activity and terminating end resection Fig. 3. The well-known phosphorylation motif binding protein 14-3-3 may inhibit the EXO1–PCNA interaction in an ATM/ATR-dependent manner (Fig. 2c)^56,57^. Taken together, these studies suggest that ATM/ATR may be involved in end resection termination through multiple different pathways. In addition to EXO1-dependent end resection termination, other groups have also reported that DNA helicase B (HELB), a DNA helicase translocates ssDNA in the 5′–3′ direction, inhibits the action of the BLM-DNA2 and EXO1 nucleases^58^. The exact mechanism by which HELB functions remains to be determined (Fig. 2d).

Recently, Ilya Finkelstein’s group reported that the RPA complex has functions in end resection regulation^59^. The unphosphorylated RPA complex stimulates the initiation of the BLM-EXO1 and BLM-DNA2 resectosomes and promotes rapid, progressive DNA resection^60^. However, phosphorylated RPA32 (pRPA32) drastically slows both BLM-EXO1 and BLM-DNA2 resectosomes and stimulates BLM strand switching when the nuclease is omitted from the reaction^60^. Moreover, BLM-EXO1 and BLM-DNA2 can resect past nucleosomes in the presence of the RPA complex but are blocked when pRPA32 is added to the in vitro reaction system. These findings imply that the RPA complex may stimulate extensive resection early, whereas increased ATM/ATR-mediated RPA complex phosphorylation terminates end resection at a later time point. However, the authors use a heavily phosphorylated RPA32 protein in their in vitro system, (Fig. 2e). End resection termination mediated by phosphorylated RPA needs further evaluation in vivo.

Interestingly, some papers also show that RNAs play roles in end resection termination. DNA damage-induced long noncoding RNAs (dilncRNAs) may block RPA complex recruitment to ssDNA by forming DNA:RNA hybrids^61^. Exosome component 10, an exosome catalytic subunit, decreases dilncRNA and DNA–RNA hybrid levels, which may facilitate RPA complex recruitment^62^. However, it is unclear whether RNA-mediated end resection termination is a universal mechanism in cells or if whether it happens at specific regions, such as transcriptively active regions (Fig. 2f).

In addition to these resectosome proteins, some NHEJ proteins also have functions in end resection termination. Several publications show that the shieldin complex is important to inhibit end resection. The shieldin complex consists of four subunits: SHLD3, REV7, SHLD2, and SHLD1^63^. Similar to RPA70, SHLD2 is also an oligonucleotide/oligosaccharide fold domain-containing protein that attenuates end resection by competing with the RPA complex for ssDNA binding and blocks end resection-related nuclease or helicase recruitment (Fig. 2g)^64,65^.

## DNA end resection and DNA replication

DNA end resection is important for precise DNA replication. ssDNA produced by end resection at stalled replication forks activates the RPA-ATR-CHK1 checkpoint and arrests the cell cycle, which is important for fork remodeling and restart. Stalled replication forks are also sensitive to nucleases involved in end resection. Stabilization of stalled replication forks prevents them from collapsing into noxious DSBs, which increases their chance for recovery. Multiple HR factors were reported to regulate the stabilization of replication forks and prevent excess resection at stalled forks, suggesting a tight link between replication fork remodeling and degradation^66^. In particular, BRCA1 and BRCA2, as well as RAD51, are suggested to be the main regulators for protecting the replication fork from nuclease-mediated degradation.

During replication stress, ssDNA is covered by the RPA complex. The RPA complex promotes replication fork remodeling from three-way junctions to four-way junctions, called fork reversal. Fork reversal helps repair collapsed replication forks and restart stalled replication forks, increasing genomic stability. After a fork is remodeled, the junction of the reversed fork can be targeted by structure-dependent endonucleases^67^. Studies in yeast and human cells suggest that MRE11 has the key role in the processing and restarting of stalled replication forks^68^. However, end resection also has a “dark side” that is evident at stalled replication forks. Unrestricted end resection by MRE11 and EXO1 can initiate the degradation of stalled forks, leading to fork collapse^69,70^. Several mechanisms have been shown to prevent abnormal degradation of reversed strands. Saravanabhavan et al. reported that DNA2 and WRN function together to degrade reversed replication forks with 5′-to-3′ polarity and promote replication restart, thus preventing aberrant processing of unresolved replication intermediates. In addition, ATP-dependent DNA helicase Q1 limits DNA2 activity by preventing extensive nascent strand degradation^71^. RAD51 has a key role in fork stabilization. RAD51 requires mediator proteins such as BRCA1 and BRCA2 to access the RPA-ssDNA complex^72^. Recruited BRCA–RAD51 promotes fork reversal formation and protects reversed replication forks from MRE11-, EXO1-, and MUS81-mediated nucleolytic degradation^66^. However, excessive RAD51 activity slows replication and causes replication forks to stall. David Cortez’s group showed that RAD51 antagonist on the X chromosome (RADX) prevents MUS81-dependent replication fork collapse. Furthermore, RADX competes with RAD51 and prevents excessive replication fork remodeling by antagonizing RAD51^73,74^. Other factors, such as the biorientation of chromosomes in cell division protein 1-like 1 (BOD1L), protect reversed forks from DNA2-mediated degradation^75^. Loss of BOD1L confers exquisite cellular sensitivity to replication stress and uncontrolled resection of damaged replication forks because of a failure to stabilize RAD51 at the replication forks. However, no study has shown that BOD1L functions in HR repair. MRN complex-interacting protein (MRNIP), an MRE11 interaction protein, was also reported to function as a replication protector by directly binding with MRE11 and suppressing the exonuclease activity of MRE11 on replication forks^76^. In contrast to BOD1L, MRNIP reportedly affects the MRN complex-mediated DSB response^77^. However, it is still not clear whether MRNIP affects end resection in HR repair.

In addition to end resection regulators in HR, NHEJ regulators also show functions in DNA replication. In DSB repair, RIF1 functions together with PTIP and the shieldin complex to promote NHEJ repair by inhibiting DNA end resection. Chirantani et al. reported that RIF1-knockout cells show elevated replication degradation and defective fork restart, which further contribute to genome instability^78^. Moreover, Lu et al. found that the CST complex (CTC1-STN1-TEN1), a downstream effector of RIF1 in end resection suppression, localizes at stalled replication forks to protect the forks from MRE11-mediated degradation under DNA replication stress^79^. CST complex deficiency leads to nascent DNA strand degradation, ssDNA accumulation at stalled replication forks and decay during replication restart. In contrast to RIF1 and the CST complex, another end resection antagonist, PTIP, was suggested to play an opposite role in replication fork stability^80^. PTIP promotes the localization of MRE11 to stalled replication forks in BRCAness (BRCA1/2 deficient) cells, thus promoting MRE11-mediated nascent DNA strand degradation and replication fork collapse. Loss of PTIP rescues the lethality of fork-stalling compounds in BRCA-deficient cells by stabilizing and remodeling stalled replication forks^80^. This study strongly suggests that the stability of nascent DNA strands can confer drug resistance to chemotherapies in BRCA-deficient cancers, indicating their great importance for cancer therapeutics.

Overall, regulated end resection at the reversed replication fork is beneficial for replication restart by inducing recombination. However, resected ends of reversed replication forks can also form a platform for the recruitment of DSB proteins. These proteins can interfere with replication fork stability, and restoration leads to chromosomal instability through uncontrolled recombination events. Therefore, end resection factors at the reversed fork need to be tightly regulated for proper fork restart and genomic stability.

## DNA end resection and DSB repair pathway choice

HR and NHEJ are the two main DSB repair pathways. At present, the pathway choice between HR and NHEJ is of particular interest in the DNA damage field. Part of this choice is dependent on the cell cycle. HR occurs in the S and G2 phases because it needs a template that is provided by the sister chromatin. Conversely, NHEJ can occur throughout interphase. In the current model, BRCA1 and 53BP1 play important roles in repair choice. BRCA1 interacts with CtIP following CDK-mediated S372 phosphorylation^7,81^. In fact, phosphorylation of CtIP is required for BRCA1 interaction but is dispensable for RPA and RAD51 foci formation in DT40 cells, implying that CtIP-dependent resection does not require interaction with BRCA1^82^. However, cells with a mutated CtIP phosphorylation site still show hypersensitivity to camptothecin and etoposide^83^. Later, Pablo Huertas’s group proved that CtIP is able to initiate end resection in the absence of BRCA1^81^. BRCA1-CtIP also suppresses the recruitment of RIF1, an essential NHEJ regulator, to DNA damage sites^84,85^. However, the mechanism for this is still unclear. In addition, our group reported that UHRF1, a ubiquitin E3 ligase, inhibits RIF1 recruitment in a BRCA1-dependent manner. UHRF1 is recruited to DSB sites through the BRCT domain of BRCA1. UHRF1-mediated RIF1 ubiquitination disrupts the 53BP1–RIF1 interaction, thus inhibiting NHEJ and promoting end resection^86^.

In addition to BRCA1/CtIP, some proteins were also reported to regulate DSB repair choice through the initiation of MRE11-mediated end resection initiation. Dynein light chain LC8 type 1 (DYNLL1) is one protein recently reported to promote NHEJ and inhibit HR^87^. DYNLL1 was shown to physically interact with MRE11 and inhibit end resection in the presence of MRN, EXO1, DNA2, and BLM in a cell-free system. However, other publications showed that DYNLL1 promotes NHEJ by promoting 53BP1 oligomerization and loading to DSB sites^88,89^. The proteasomal shuttle factor ubiquilin 4 (UBQLN4) was also reported to regulate DSB repair choice through MRE11^90^. Upon DNA damage, UBQLN4 is phosphorylated by ATM and interacts with ubiquitylated MRE11 to promote MRE11 degradation. UBQLN4 deficiency causes chromatin retention of MRE11, enhancing nonphysiological HR activity. In contrast, UBQLN4 overexpression represses HR and promotes NHEJ^90^. Moreover, UBQLN4 is overexpressed in aggressive tumors, leading to deficient HR and conferring sensitivity to PARP inhibitor treatment.

53BP1 works as an antagonist of BRCA1 and has a key role in DSB repair choice. 53BP1 promotes classical non-homologous end joining (c-NHEJ) by recruiting RIF1 to inhibit BRCA1 recruitment to break sites, thereby antagonizing BRCA1-CtIP-mediated end resection and blocking BRCA2/RAD51-mediated homologous recombination^91^. RIF1 has been shown to inhibit BRCA1 recruitment in the G1 phase, although the mechanism is unclear. This may be one reason that HR is restricted to the S and G2 phase^85^. In addition to its role in “pre-resection” regulation for repair choice, RIF1 has been reported in recent publications to function in a “post-resection” step by recruiting the shieldin complex to resected DNA ends^92^. Within the shieldin complex, SHLD3 directly interacts with RIF1 and facilitates the recruitment of REV7, which is an adaptor protein that recruits SHLD2^64^. SHLD2 contains three tandem OB-fold domains that have ssDNA-binding activity. SHLD2 binds to a ssDNA end and protects the ssDNA end from extensive resection by EXO1/DNA2 and consequently inhibits HR^63^. The knockdown of REV7 or SHLD2 restores PARP inhibitor resistance in BRCA1-deficient cells^93^. The function of SHLD1 is still not clear. It has been shown that knocking down SHLD1 decreases SHLD2 foci, implying that SHLD1 may stabilize SHLD2 on ssDNA. In addition to protecting the ssDNA end, the shieldin complex was reported by Callen et al. to inhibit RNF168-mediated PALB2-RAD51 loading to DSB sites in *BRCA1*^*Δ11*^*53BP1*^*S25A*^ cells (S25A abolishes the PTIP interaction), which suggests that the shieldin complex has dual functions in antagonizing HR^94^. Recently, one publication suggested that TRIP13, an ATPase, functions as an antagonist to the shieldin complex and promotes HR. TRIP13 physically interacts with REV7 and inhibits NHEJ by changing the closed conformation of REV7 to an open conformation, which disrupts the shieldin complex^93^. Similar to the shieldin complex, the CST complex is a novel end resection regulator that also functions in DSB repair choice. The mechanism involves CST recruiting DNA polymerase α (polα) to fill in the resected 3′ single-stranded DNA end^95^. Titia de Lange’s group concluded that CST/polα is a downstream effector of the shieldin complex because CST can be recruited by 53BP1 or SHLD2^96^. CST mainly functions at telomeric regions^97^. However, Sven Rottenberg’s group thinks that CST/polα is an alternative pathway to shieldin complex activity and functions at nontelomeric regions^98^. CTC1 is also an OB-fold domain-containing protein, which is similar to RPA1 and SHLD2, and CTC1 is capable of binding to ssDNA independently of SHLD2^99^. Thus, they presumed that the three complexes (RPA, shieldin, CST) likely compete at the 3′ single-stranded DNA end. However, some researchers have suggested that polα only has limited DNA polymerase activity, which might be insufficient to process ssDNA ends^64^. The detailed mechanism for this regulation still needs further study.

In addition to the shieldin and the CST complex, deletion of PTIP also shows increased DNA end resection, suggesting that PTIP is an antagonist to DNA end resection. However, the distinct role of PTIP and the shieldin/CST complex in DNA end resection attenuation is not very clear. Callen et al. suggested that PTIP preferentially inhibits DNA2-mediated end resection, whereas the shieldin complex prefers to inhibit EXO1-mediated end resection. In this model, the two function independently while also complementing each other^94^. The PTIP-associated DNA exo/endonuclease Artemis is also reported to function in DSB repair choice. Artemis shows both 5′–3′ exonuclease and endonuclease activity in vitro. Independent of RIF1, Artemis is recruited by 53BP1-PTIP following DNA damage and trims the resected ssDNA with its endonuclease activity, thus promoting NHEJ^100^.

## Concluding remarks

As end resection has an essential role in DSB repair, it also shows significant clinical relevance in cancer therapy. Cancer cells with a mutation or deficiency of a core end resection-regulating gene are more sensitive to radiotherapy and chemotherapy. However, in BRCA1-deficient patients, loss of important end resection antagonists, such as 53BP1, RIF1, and the shieldin complex, leads to enhanced resistance to PARP inhibitor treatment due to the recovery of end resection. Thus, the status of end resection suggests its importance for guiding cancer therapy. Because of its critical role in DNA repair and cancer therapeutics, the mechanism for end resection has been thoroughly studied in the past decade. However, there are still many unanswered questions, several of which we highlight here:

- Because of the toxicity induced by hyperresection in cells, how is the termination of end resection determined under physiological conditions?
- What are the downstream effectors of 53BP1-RIF1-shieldin that contribute to the inhibition of end resection? As these proteins have no enzymatic activity, how is the resected ssDNA end processed?
- What is the role of the shieldin complex, an important antagonist of end resection in DSB repair, in DNA replication?

The answers to these questions will further broaden our current view of end resection, DNA repair, and cancer therapeutics.
